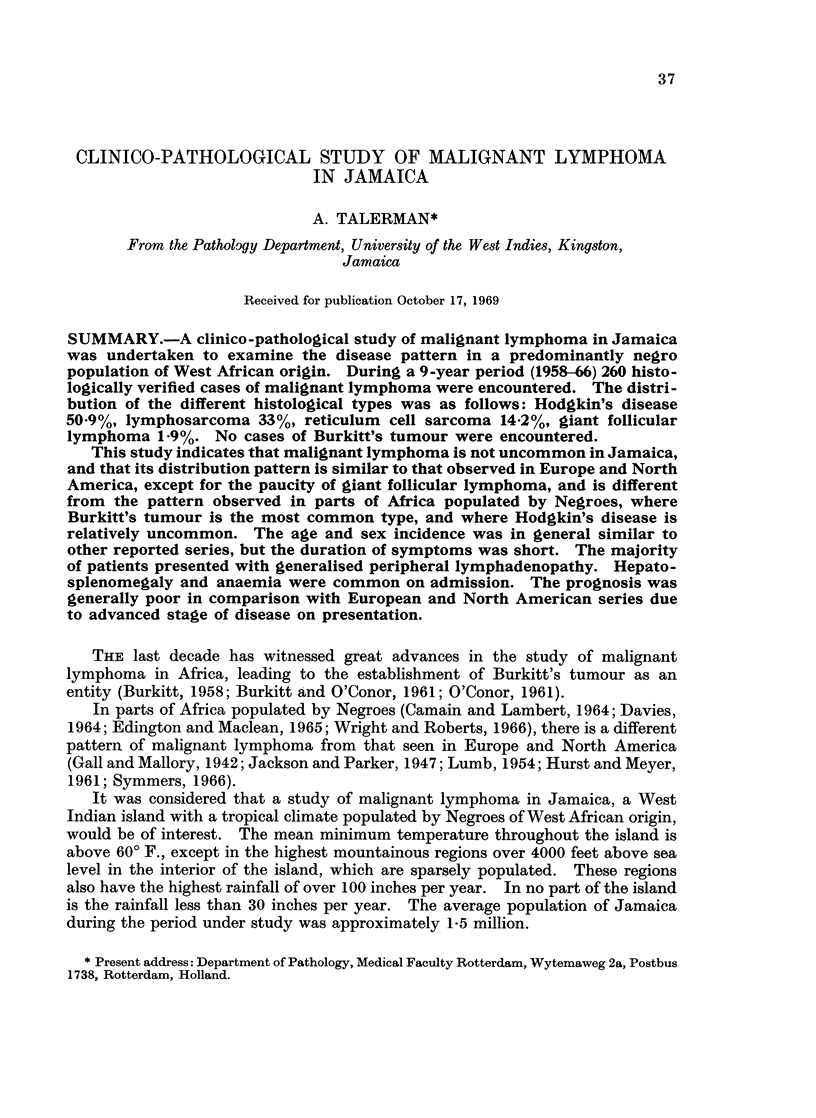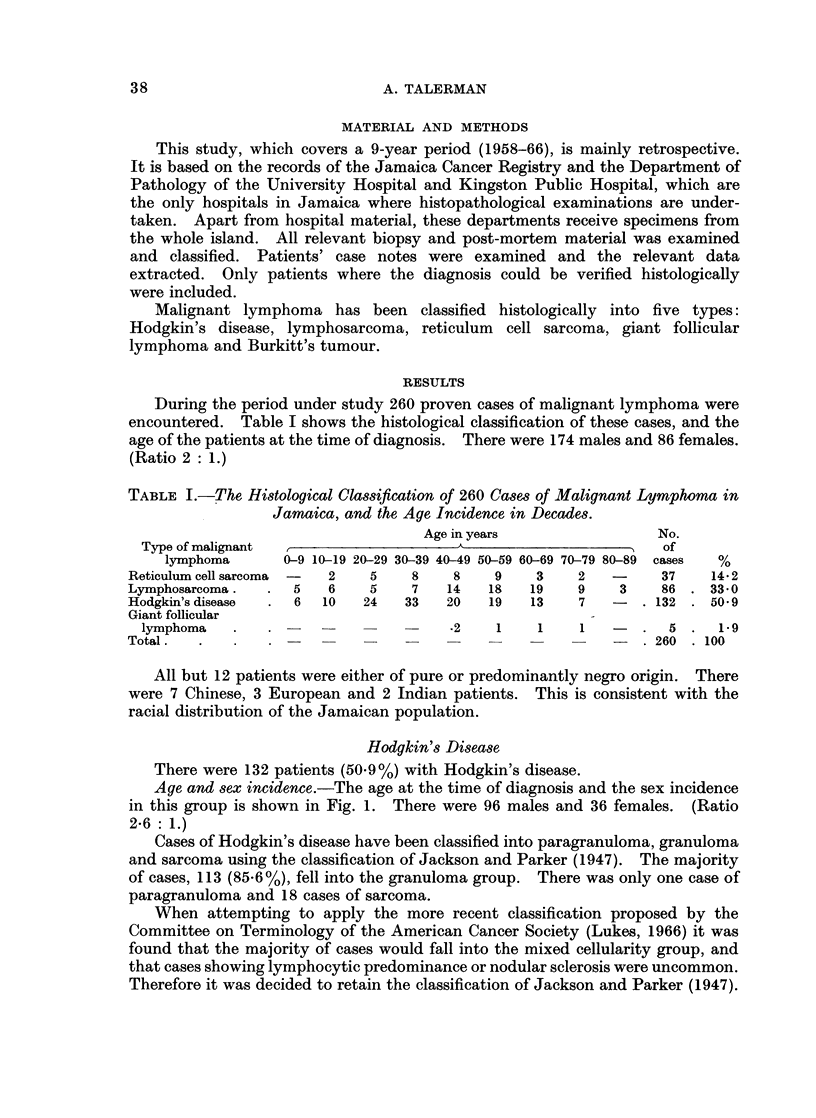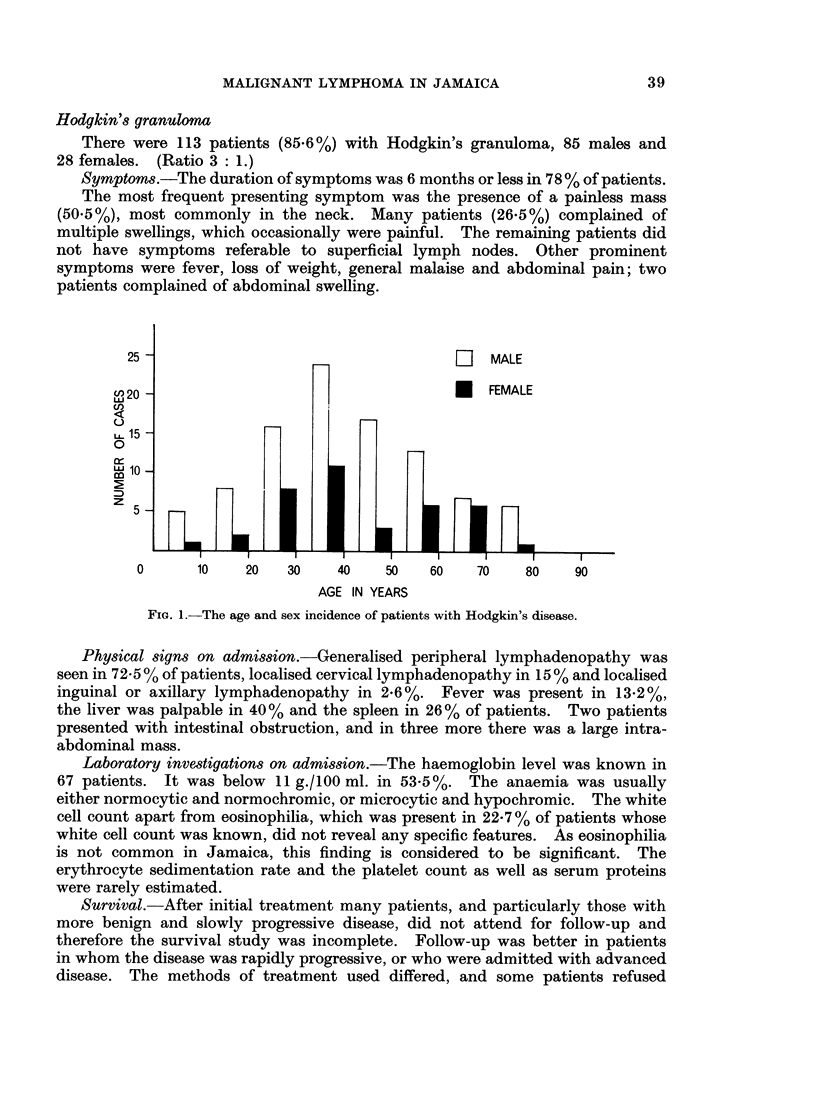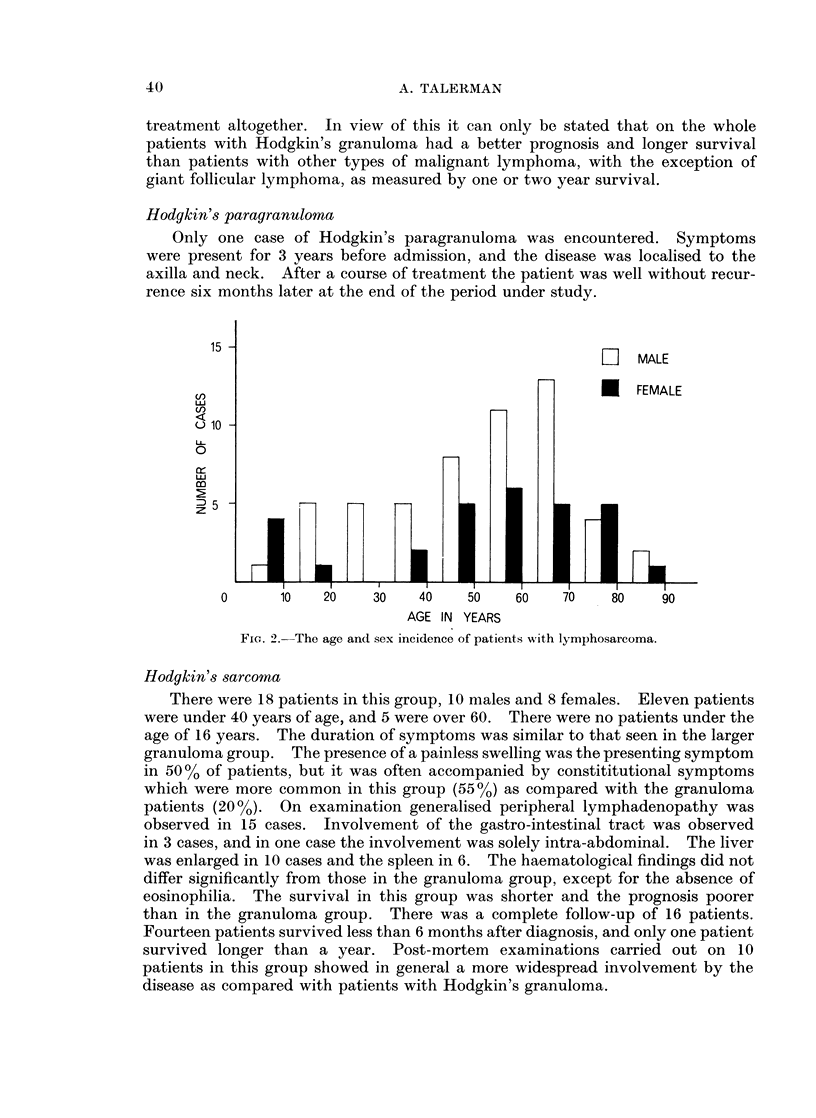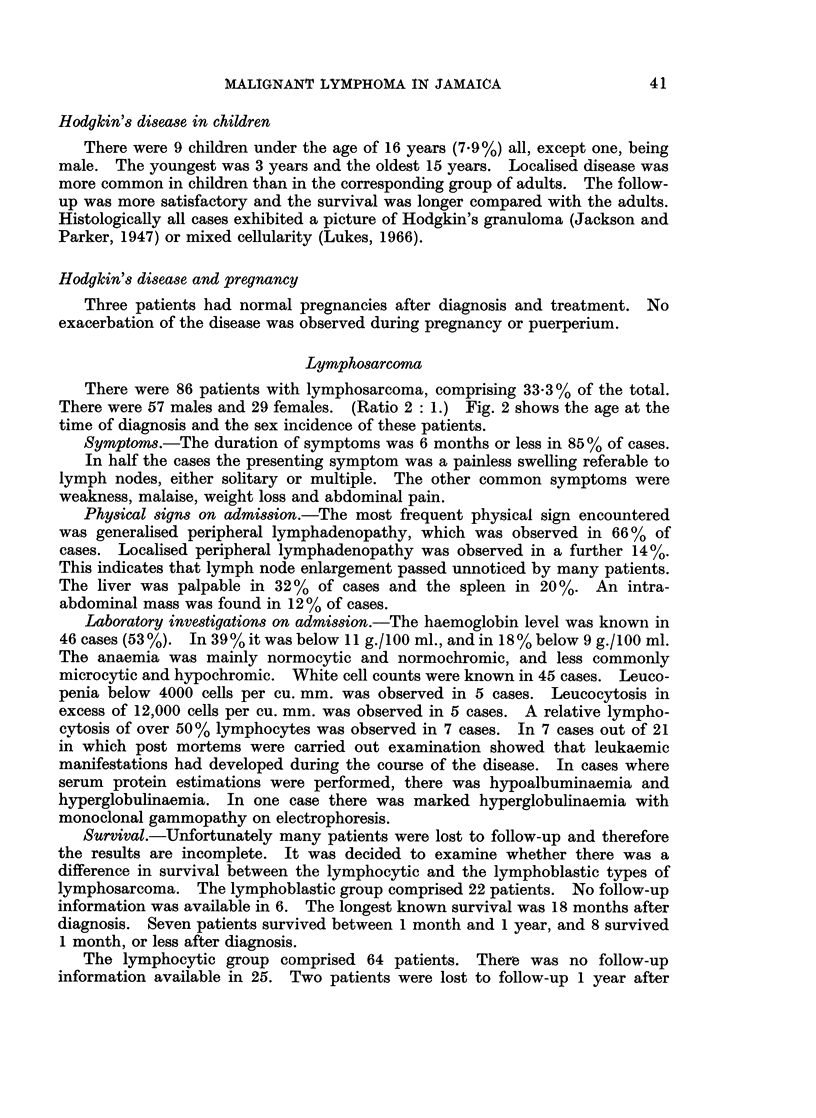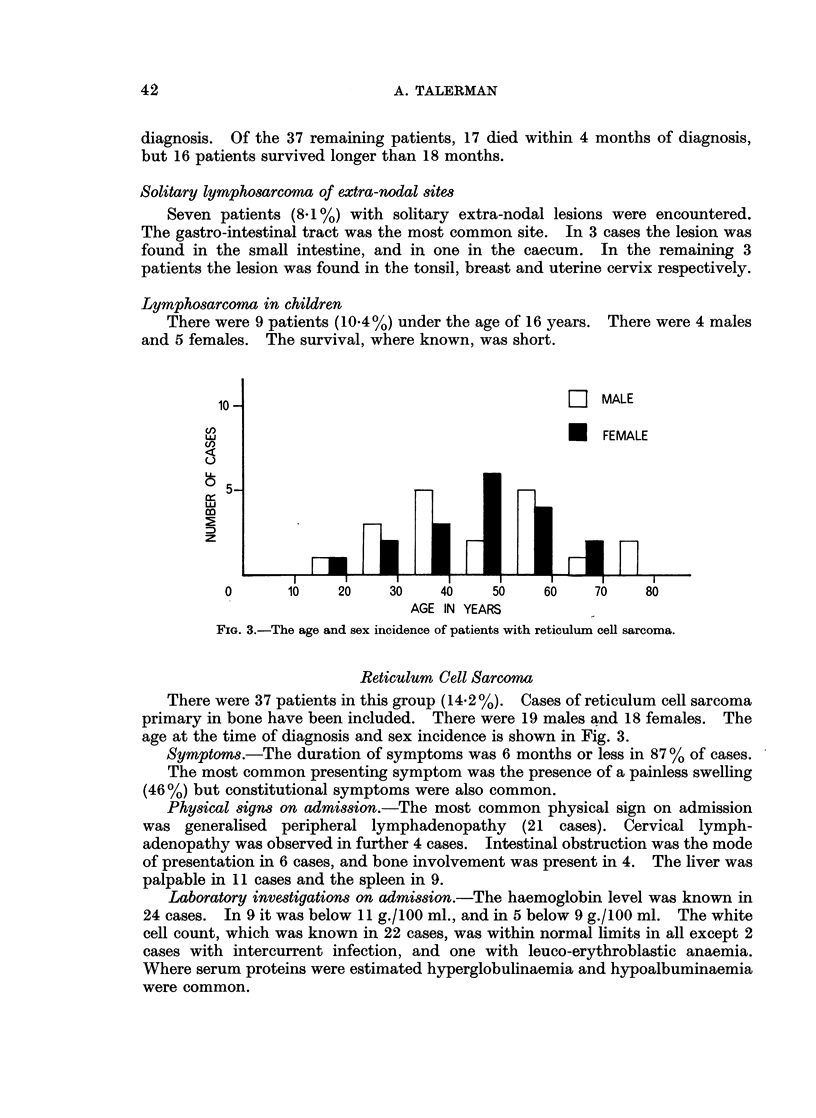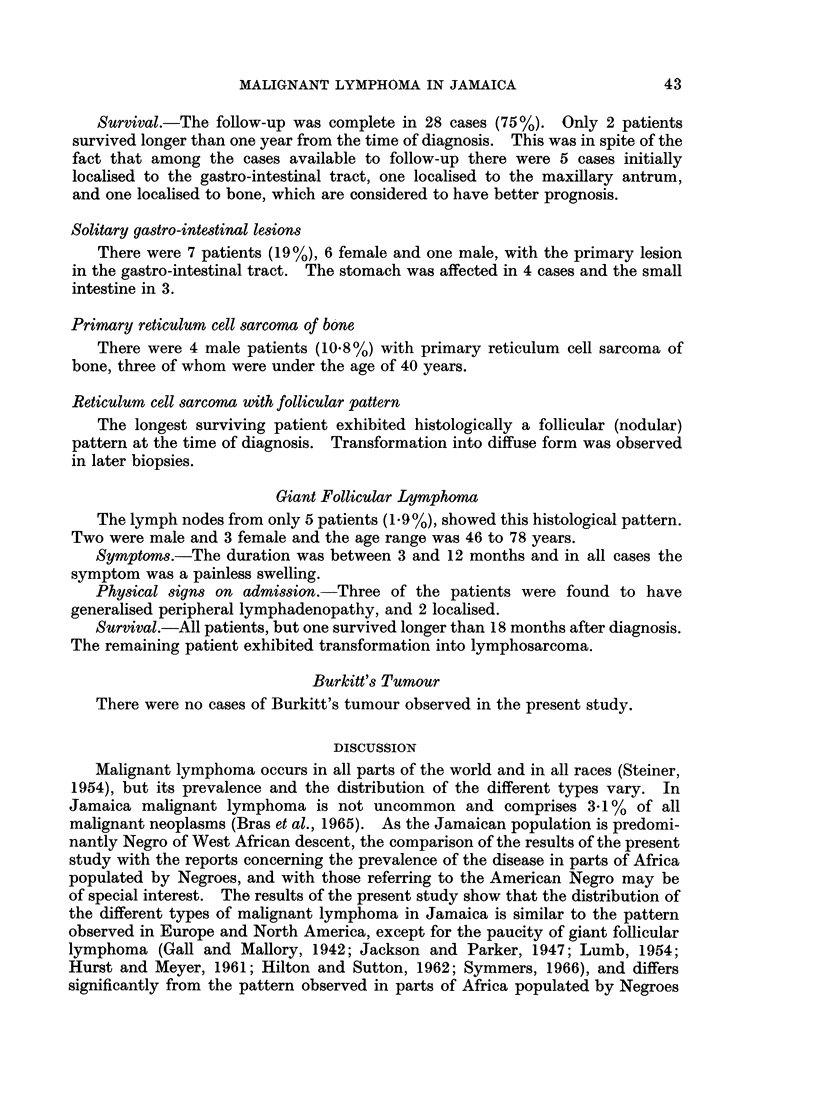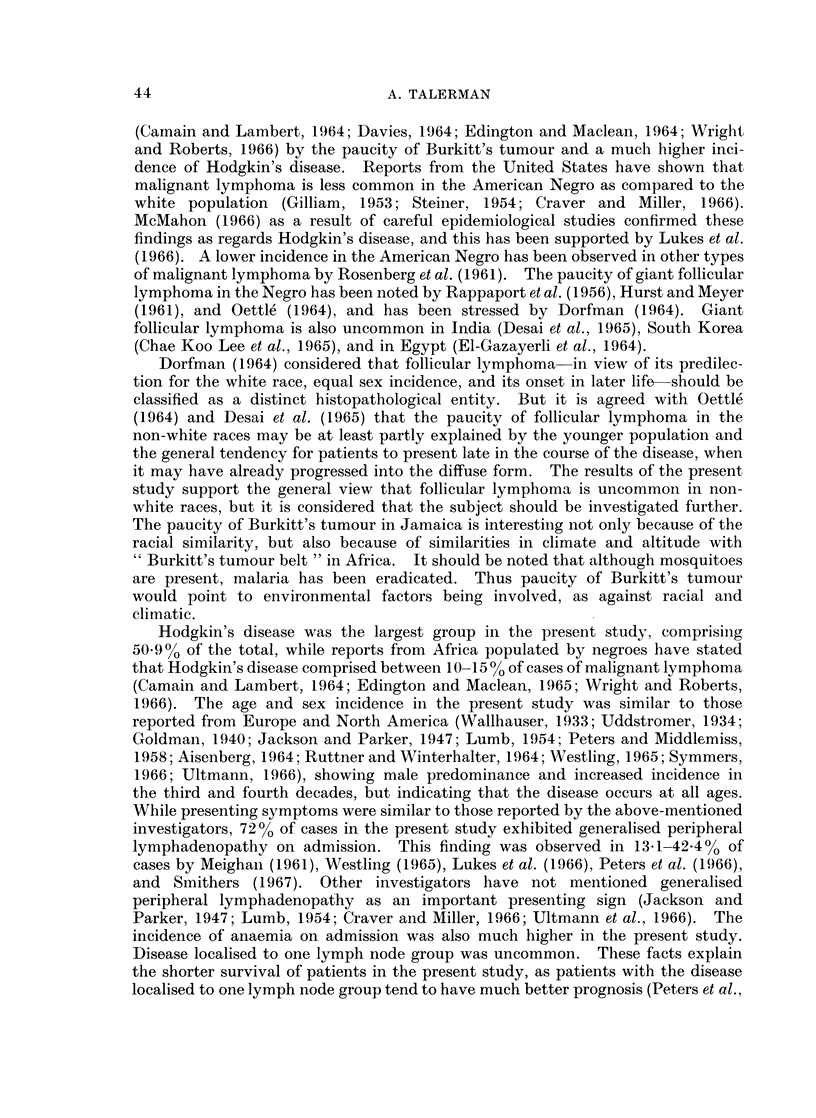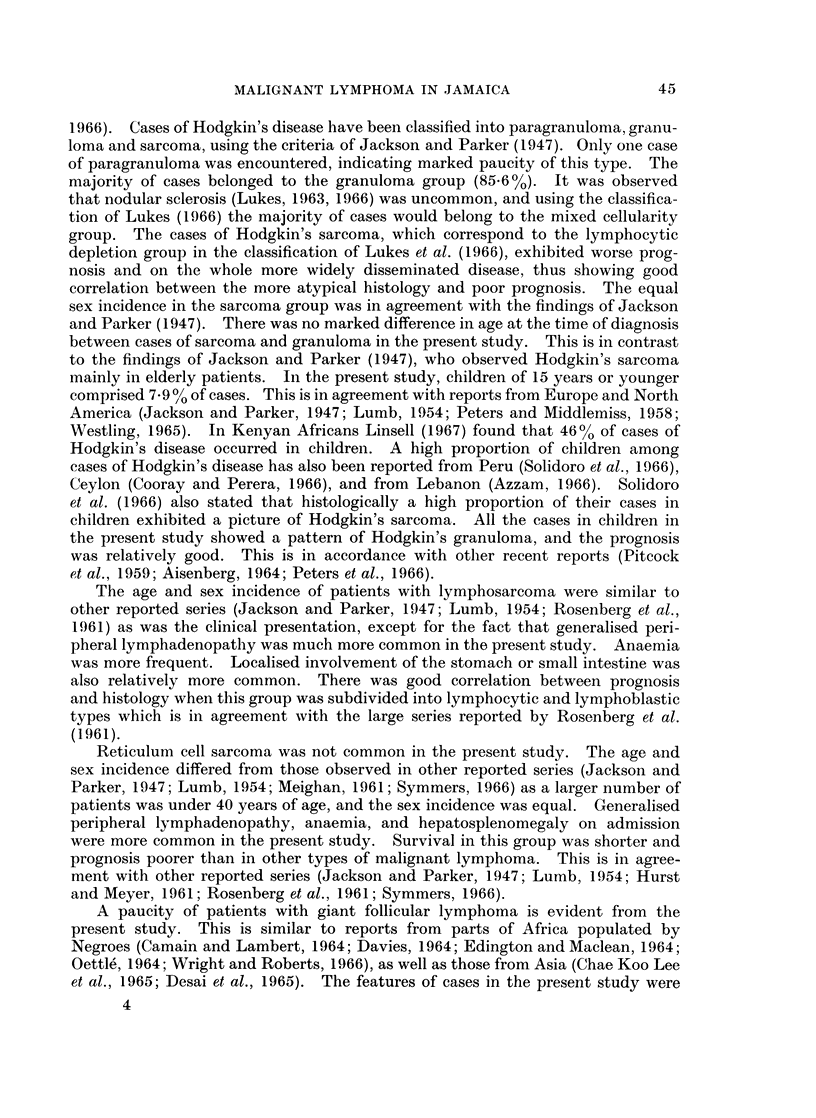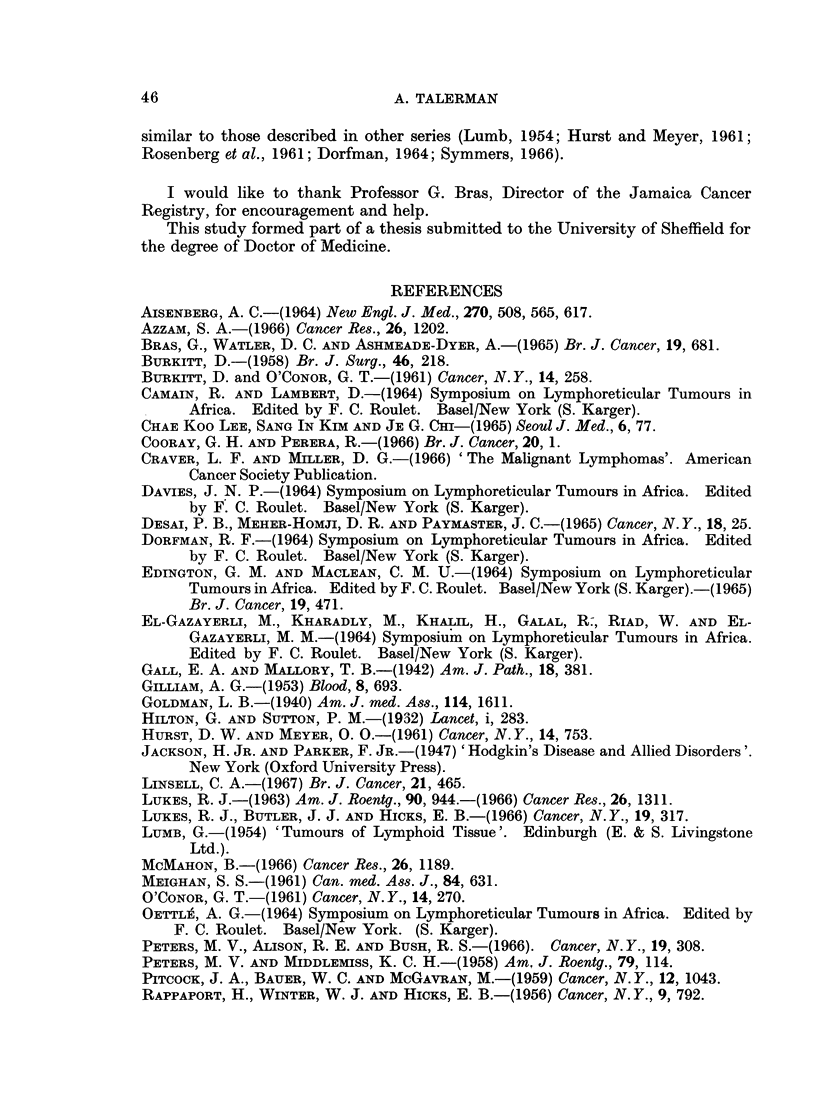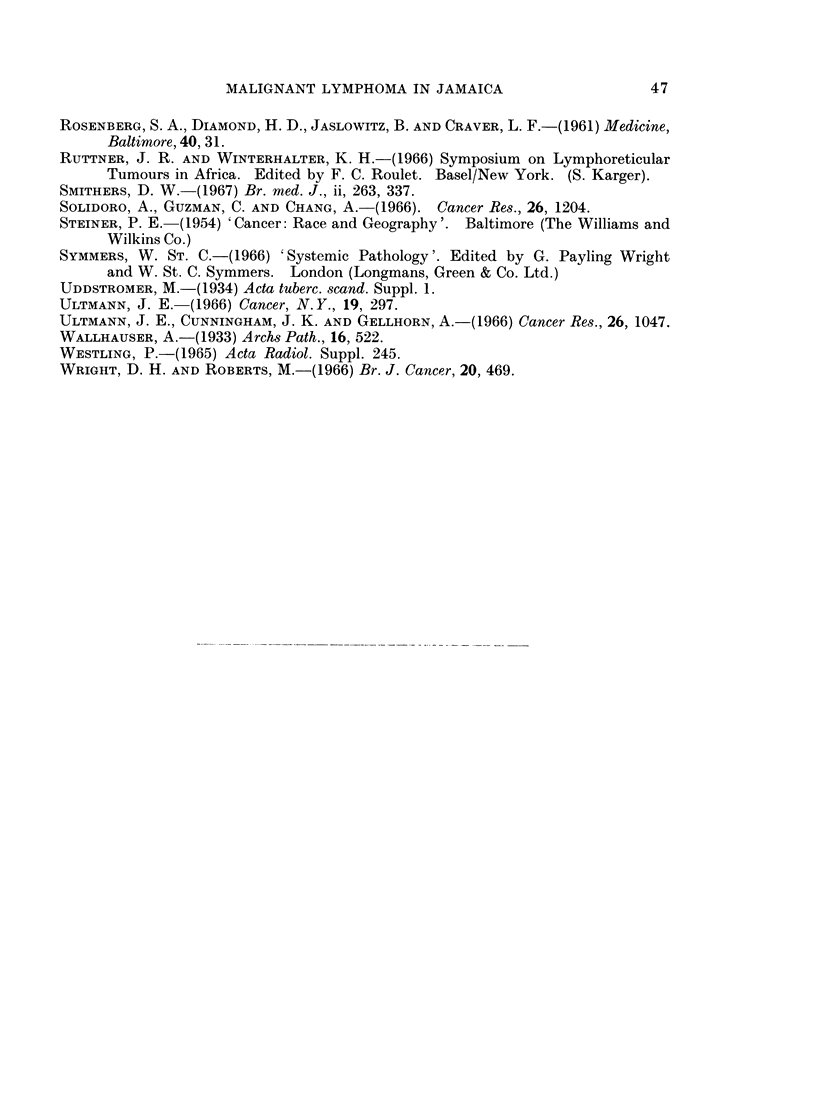# Clinico-Pathological Study of Malignant Lymphoma in Jamaica

**DOI:** 10.1038/bjc.1970.6

**Published:** 1970-03

**Authors:** A. Talerman

## Abstract

A clinico-pathological study of malignant lymphoma in Jamaica was undertaken to examine the disease pattern in a predominantly negro population of West African origin. During a 9-year period (1958-66) 260 histologically verified cases of malignant lymphoma were encountered. The distribution of the different histological types was as follows: Hodgkin's disease 50.9%, lymphosarcoma 33%, reticulum cell sarcoma 14.2%, giant follicular lymphoma 1.9%. No cases of Burkitt's tumour were encountered.

This study indicates that malignant lymphoma is not uncommon in Jamaica, and that its distribution pattern is similar to that observed in Europe and North America, except for the paucity of giant follicular lymphoma, and is different from the pattern observed in parts of Africa populated by Negroes, where Burkitt's tumour is the most common type, and where Hodgkin's disease is relatively uncommon. The age and sex incidence was in general similar to other reported series, but the duration of symptoms was short. The majority of patients presented with generalised peripheral lymphadenopathy. Hepatosplenomegaly and anaemia were common on admission. The prognosis was generally poor in comparison with European and North American series due to advanced stage of disease on presentation.


### CLINICO-PATHOLOGICAL STUDY OF MALIGNANT LYMPHOMA IN JAMAICA

A. TALERMAN*

*From the Pathology Department, University of the West Indies, Kingston, Jamaica*

Received for publication October 17, 1969

**SUMMARY.—A clinico-pathological study of malignant lymphoma in Jamaica was undertaken to examine the disease pattern in a predominantly negro population of West African origin. During a 9-year period (1958–66) 260 histologically verified cases of malignant lymphoma were encountered. The distribution of the different histological types was as follows: Hodgkin's disease 50·9%, lymphosarcoma 33%, reticulum cell sarcoma 14·2%, giant follicular lymphoma 1·9%. No cases of Burkitt's tumour were encountered.**

**This study indicates that malignant lymphoma is not uncommon in Jamaica, and that its distribution pattern is similar to that observed in Europe and North America, except for the paucity of giant follicular lymphoma, and is different from the pattern observed in parts of Africa populated by Negroes, where Burkitt's tumour is the most common type, and where Hodgkin's disease is relatively uncommon. The age and sex incidence was in general similar to other reported series, but the duration of symptoms was short. The majority of patients presented with generalised peripheral lymphadenopathy. Hepatosplenomegaly and anaemia were common on admission. The prognosis was generally poor in comparison with European and North American series due to advanced stage of disease on presentation.**

THE last decade has witnessed great advances in the study of malignant lymphoma in Africa, leading to the establishment of Burkitt's tumour as an entity (Burkitt, 1958; Burkitt and O'Conor, 1961; O'Conor, 1961).

In parts of Africa populated by Negroes (Camain and Lambert, 1964; Davies, 1964; Edington and Maclean, 1965; Wright and Roberts, 1966), there is a different pattern of malignant lymphoma from that seen in Europe and North America (Gall and Mallory, 1942; Jackson and Parker, 1947; Lumb, 1954; Hurst and Meyer, 1961; Symmers, 1966).

It was considered that a study of malignant lymphoma in Jamaica, a West Indian island with a tropical climate populated by Negroes of West African origin, would be of interest. The mean minimum temperature throughout the island is above 60° F., except in the highest mountainous regions over 4000 feet above sea level in the interior of the island, which are sparsely populated. These regions also have the highest rainfall of over 100 inches per year. In no part of the island is the rainfall less than 30 inches per year. The average population of Jamaica during the period under study was approximately 1·5 million.

* Present address: Department of Pathology, Medical Faculty Rotterdam, Wytemaweg 2a, Postbus 1738, Rotterdam, Holland.

### MATERIAL AND METHODS

This study, which covers a 9-year period (1958–66), is mainly retrospective. It is based on the records of the Jamaica Cancer Registry and the Department of Pathology of the University Hospital and Kingston Public Hospital, which are the only hospitals in Jamaica where histopathological examinations are undertaken. Apart from hospital material, these departments receive specimens from the whole island. All relevant biopsy and post-mortem material was examined and classified. Patients' case notes were examined and the relevant data extracted. Only patients where the diagnosis could be verified histologically were included.

Malignant lymphoma has been classified histologically into five types: Hodgkin's disease, lymphosarcoma, reticulum cell sarcoma, giant follicular lymphoma and Burkitt's tumour.

### RESULTS

During the period under study 260 proven cases of malignant lymphoma were encountered. Table I shows the histological classification of these cases, and the age of the patients at the time of diagnosis. There were 174 males and 86 females. (Ratio 2 : 1.)

TABLE I.—*The Histological Classification of* 260 *Cases of Malignant Lymphoma in Jamaica, and the Age Incidence in Decades.*

| Type of malignant lymphoma | Age in years | | | | | | | | | No. of cases | % |
|---|---|---|---|---|---|---|---|---|---|---|---|
| | 0–9 | 10–19 | 20–29 | 30–39 | 40–49 | 50–59 | 60–69 | 70–79 | 80–89 | | |
| Reticulum cell sarcoma | — | 2 | 5 | 8 | 8 | 9 | 3 | 2 | — | 37 | 14·2 |
| Lymphosarcoma | 5 | 6 | 5 | 7 | 14 | 18 | 19 | 9 | 3 | 86 | 33·0 |
| Hodgkin's disease | 6 | 10 | 24 | 33 | 20 | 19 | 13 | 7 | — | 132 | 50·9 |
| Giant follicular lymphoma | — | — | — | — | ·2 | 1 | 1 | 1 | — | 5 | 1·9 |
| Total | — | — | — | — | — | — | — | — | — | 260 | 100 |

All but 12 patients were either of pure or predominantly negro origin. There were 7 Chinese, 3 European and 2 Indian patients. This is consistent with the racial distribution of the Jamaican population.

### *Hodgkin's Disease*

There were 132 patients (50·9%) with Hodgkin's disease.

*Age and sex incidence.*—The age at the time of diagnosis and the sex incidence in this group is shown in Fig. 1. There were 96 males and 36 females. (Ratio 2·6 : 1.)

Cases of Hodgkin's disease have been classified into paragranuloma, granuloma and sarcoma using the classification of Jackson and Parker (1947). The majority of cases, 113 (85·6%), fell into the granuloma group. There was only one case of paragranuloma and 18 cases of sarcoma.

When attempting to apply the more recent classification proposed by the Committee on Terminology of the American Cancer Society (Lukes, 1966) it was found that the majority of cases would fall into the mixed cellularity group, and that cases showing lymphocytic predominance or nodular sclerosis were uncommon. Therefore it was decided to retain the classification of Jackson and Parker (1947).

*Hodgkin's granuloma*

There were 113 patients (85·6%) with Hodgkin's granuloma, 85 males and 28 females. (Ratio 3 : 1.)

*Symptoms.*—The duration of symptoms was 6 months or less in 78% of patients. The most frequent presenting symptom was the presence of a painless mass (50·5%), most commonly in the neck. Many patients (26·5%) complained of multiple swellings, which occasionally were painful. The remaining patients did not have symptoms referable to superficial lymph nodes. Other prominent symptoms were fever, loss of weight, general malaise and abdominal pain; two patients complained of abdominal swelling.

FIG. 1.—The age and sex incidence of patients with Hodgkin's disease.

*Physical signs on admission.*—Generalised peripheral lymphadenopathy was seen in 72·5% of patients, localised cervical lymphadenopathy in 15% and localised inguinal or axillary lymphadenopathy in 2·6%. Fever was present in 13·2%, the liver was palpable in 40% and the spleen in 26% of patients. Two patients presented with intestinal obstruction, and in three more there was a large intra-abdominal mass.

*Laboratory investigations on admission.*—The haemoglobin level was known in 67 patients. It was below 11 g./100 ml. in 53·5%. The anaemia was usually either normocytic and normochromic, or microcytic and hypochromic. The white cell count apart from eosinophilia, which was present in 22·7% of patients whose white cell count was known, did not reveal any specific features. As eosinophilia is not common in Jamaica, this finding is considered to be significant. The erythrocyte sedimentation rate and the platelet count as well as serum proteins were rarely estimated.

*Survival.*—After initial treatment many patients, and particularly those with more benign and slowly progressive disease, did not attend for follow-up and therefore the survival study was incomplete. Follow-up was better in patients in whom the disease was rapidly progressive, or who were admitted with advanced disease. The methods of treatment used differed, and some patients refused treatment altogether. In view of this it can only be stated that on the whole patients with Hodgkin's granuloma had a better prognosis and longer survival than patients with other types of malignant lymphoma, with the exception of giant follicular lymphoma, as measured by one or two year survival.

*Hodgkin's paragranuloma*

Only one case of Hodgkin's paragranuloma was encountered. Symptoms were present for 3 years before admission, and the disease was localised to the axilla and neck. After a course of treatment the patient was well without recurrence six months later at the end of the period under study.

FIG. 2.—The age and sex incidence of patients with lymphosarcoma.

*Hodgkin's sarcoma*

There were 18 patients in this group, 10 males and 8 females. Eleven patients were under 40 years of age, and 5 were over 60. There were no patients under the age of 16 years. The duration of symptoms was similar to that seen in the larger granuloma group. The presence of a painless swelling was the presenting symptom in 50% of patients, but it was often accompanied by constititutional symptoms which were more common in this group (55%) as compared with the granuloma patients (20%). On examination generalised peripheral lymphadenopathy was observed in 15 cases. Involvement of the gastro-intestinal tract was observed in 3 cases, and in one case the involvement was solely intra-abdominal. The liver was enlarged in 10 cases and the spleen in 6. The haematological findings did not differ significantly from those in the granuloma group, except for the absence of eosinophilia. The survival in this group was shorter and the prognosis poorer than in the granuloma group. There was a complete follow-up of 16 patients. Fourteen patients survived less than 6 months after diagnosis, and only one patient survived longer than a year. Post-mortem examinations carried out on 10 patients in this group showed in general a more widespread involvement by the disease as compared with patients with Hodgkin's granuloma.

*Hodgkin's disease in children*

There were 9 children under the age of 16 years (7·9%) all, except one, being male. The youngest was 3 years and the oldest 15 years. Localised disease was more common in children than in the corresponding group of adults. The follow-up was more satisfactory and the survival was longer compared with the adults. Histologically all cases exhibited a picture of Hodgkin's granuloma (Jackson and Parker, 1947) or mixed cellularity (Lukes, 1966).

*Hodgkin's disease and pregnancy*

Three patients had normal pregnancies after diagnosis and treatment. No exacerbation of the disease was observed during pregnancy or puerperium.

*Lymphosarcoma*

There were 86 patients with lymphosarcoma, comprising 33·3% of the total. There were 57 males and 29 females. (Ratio 2 : 1.) Fig. 2 shows the age at the time of diagnosis and the sex incidence of these patients.

*Symptoms*.—The duration of symptoms was 6 months or less in 85% of cases. In half the cases the presenting symptom was a painless swelling referable to lymph nodes, either solitary or multiple. The other common symptoms were weakness, malaise, weight loss and abdominal pain.

*Physical signs on admission*.—The most frequent physical sign encountered was generalised peripheral lymphadenopathy, which was observed in 66% of cases. Localised peripheral lymphadenopathy was observed in a further 14%. This indicates that lymph node enlargement passed unnoticed by many patients. The liver was palpable in 32% of cases and the spleen in 20%. An intra-abdominal mass was found in 12% of cases.

*Laboratory investigations on admission*.—The haemoglobin level was known in 46 cases (53%). In 39% it was below 11 g./100 ml., and in 18% below 9 g./100 ml. The anaemia was mainly normocytic and normochromic, and less commonly microcytic and hypochromic. White cell counts were known in 45 cases. Leucopenia below 4000 cells per cu. mm. was observed in 5 cases. Leucocytosis in excess of 12,000 cells per cu. mm. was observed in 5 cases. A relative lymphocytosis of over 50% lymphocytes was observed in 7 cases. In 7 cases out of 21 in which post mortems were carried out examination showed that leukaemic manifestations had developed during the course of the disease. In cases where serum protein estimations were performed, there was hypoalbuminaemia and hyperglobulinaemia. In one case there was marked hyperglobulinaemia with monoclonal gammopathy on electrophoresis.

*Survival*.—Unfortunately many patients were lost to follow-up and therefore the results are incomplete. It was decided to examine whether there was a difference in survival between the lymphocytic and the lymphoblastic types of lymphosarcoma. The lymphoblastic group comprised 22 patients. No follow-up information was available in 6. The longest known survival was 18 months after diagnosis. Seven patients survived between 1 month and 1 year, and 8 survived 1 month, or less after diagnosis.

The lymphocytic group comprised 64 patients. There was no follow-up information available in 25. Two patients were lost to follow-up 1 year after diagnosis. Of the 37 remaining patients, 17 died within 4 months of diagnosis, but 16 patients survived longer than 18 months.

*Solitary lymphosarcoma of extra-nodal sites*

Seven patients (8·1%) with solitary extra-nodal lesions were encountered. The gastro-intestinal tract was the most common site. In 3 cases the lesion was found in the small intestine, and in one in the caecum. In the remaining 3 patients the lesion was found in the tonsil, breast and uterine cervix respectively.

*Lymphosarcoma in children*

There were 9 patients (10·4%) under the age of 16 years. There were 4 males and 5 females. The survival, where known, was short.

FIG. 3.—The age and sex incidence of patients with reticulum cell sarcoma.

### *Reticulum Cell Sarcoma*

There were 37 patients in this group (14·2%). Cases of reticulum cell sarcoma primary in bone have been included. There were 19 males and 18 females. The age at the time of diagnosis and sex incidence is shown in Fig. 3.

*Symptoms.*—The duration of symptoms was 6 months or less in 87% of cases. The most common presenting symptom was the presence of a painless swelling (46%) but constitutional symptoms were also common.

*Physical signs on admission.*—The most common physical sign on admission was generalised peripheral lymphadenopathy (21 cases). Cervical lymphadenopathy was observed in further 4 cases. Intestinal obstruction was the mode of presentation in 6 cases, and bone involvement was present in 4. The liver was palpable in 11 cases and the spleen in 9.

*Laboratory investigations on admission.*—The haemoglobin level was known in 24 cases. In 9 it was below 11 g./100 ml., and in 5 below 9 g./100 ml. The white cell count, which was known in 22 cases, was within normal limits in all except 2 cases with intercurrent infection, and one with leuco-erythroblastic anaemia. Where serum proteins were estimated hyperglobulinaemia and hypoalbuminaemia were common.

*Survival.*—The follow-up was complete in 28 cases (75%). Only 2 patients survived longer than one year from the time of diagnosis. This was in spite of the fact that among the cases available to follow-up there were 5 cases initially localised to the gastro-intestinal tract, one localised to the maxillary antrum, and one localised to bone, which are considered to have better prognosis.

*Solitary gastro-intestinal lesions*

There were 7 patients (19%), 6 female and one male, with the primary lesion in the gastro-intestinal tract. The stomach was affected in 4 cases and the small intestine in 3.

*Primary reticulum cell sarcoma of bone*

There were 4 male patients (10·8%) with primary reticulum cell sarcoma of bone, three of whom were under the age of 40 years.

*Reticulum cell sarcoma with follicular pattern*

The longest surviving patient exhibited histologically a follicular (nodular) pattern at the time of diagnosis. Transformation into diffuse form was observed in later biopsies.

### *Giant Follicular Lymphoma*

The lymph nodes from only 5 patients (1·9%), showed this histological pattern. Two were male and 3 female and the age range was 46 to 78 years.

*Symptoms.*—The duration was between 3 and 12 months and in all cases the symptom was a painless swelling.

*Physical signs on admission.*—Three of the patients were found to have generalised peripheral lymphadenopathy, and 2 localised.

*Survival.*—All patients, but one survived longer than 18 months after diagnosis. The remaining patient exhibited transformation into lymphosarcoma.

### *Burkitt's Tumour*

There were no cases of Burkitt's tumour observed in the present study.

### DISCUSSION

Malignant lymphoma occurs in all parts of the world and in all races (Steiner, 1954), but its prevalence and the distribution of the different types vary. In Jamaica malignant lymphoma is not uncommon and comprises 3·1% of all malignant neoplasms (Bras *et al.*, 1965). As the Jamaican population is predominantly Negro of West African descent, the comparison of the results of the present study with the reports concerning the prevalence of the disease in parts of Africa populated by Negroes, and with those referring to the American Negro may be of special interest. The results of the present study show that the distribution of the different types of malignant lymphoma in Jamaica is similar to the pattern observed in Europe and North America, except for the paucity of giant follicular lymphoma (Gall and Mallory, 1942; Jackson and Parker, 1947; Lumb, 1954; Hurst and Meyer, 1961; Hilton and Sutton, 1962; Symmers, 1966), and differs significantly from the pattern observed in parts of Africa populated by Negroes

(Camain and Lambert, 1964; Davies, 1964; Edington and Maclean, 1964; Wright and Roberts, 1966) by the paucity of Burkitt's tumour and a much higher incidence of Hodgkin's disease. Reports from the United States have shown that malignant lymphoma is less common in the American Negro as compared to the white population (Gilliam, 1953; Steiner, 1954; Craver and Miller, 1966). McMahon (1966) as a result of careful epidemiological studies confirmed these findings as regards Hodgkin's disease, and this has been supported by Lukes *et al.* (1966). A lower incidence in the American Negro has been observed in other types of malignant lymphoma by Rosenberg *et al.* (1961). The paucity of giant follicular lymphoma in the Negro has been noted by Rappaport *et al.* (1956), Hurst and Meyer (1961), and Oettlé (1964), and has been stressed by Dorfman (1964). Giant follicular lymphoma is also uncommon in India (Desai *et al.*, 1965), South Korea (Chae Koo Lee *et al.*, 1965), and in Egypt (El-Gazayerli *et al.*, 1964).

Dorfman (1964) considered that follicular lymphoma—in view of its predilection for the white race, equal sex incidence, and its onset in later life—should be classified as a distinct histopathological entity. But it is agreed with Oettlé (1964) and Desai *et al.* (1965) that the paucity of follicular lymphoma in the non-white races may be at least partly explained by the younger population and the general tendency for patients to present late in the course of the disease, when it may have already progressed into the diffuse form. The results of the present study support the general view that follicular lymphoma is uncommon in non-white races, but it is considered that the subject should be investigated further. The paucity of Burkitt's tumour in Jamaica is interesting not only because of the racial similarity, but also because of similarities in climate and altitude with " Burkitt's tumour belt " in Africa. It should be noted that although mosquitoes are present, malaria has been eradicated. Thus paucity of Burkitt's tumour would point to environmental factors being involved, as against racial and climatic.

Hodgkin's disease was the largest group in the present study, comprising 50·9% of the total, while reports from Africa populated by negroes have stated that Hodgkin's disease comprised between 10–15% of cases of malignant lymphoma (Camain and Lambert, 1964; Edington and Maclean, 1965; Wright and Roberts, 1966). The age and sex incidence in the present study was similar to those reported from Europe and North America (Wallhauser, 1933; Uddstromer, 1934; Goldman, 1940; Jackson and Parker, 1947; Lumb, 1954; Peters and Middlemiss, 1958; Aisenberg, 1964; Ruttner and Winterhalter, 1964; Westling, 1965; Symmers, 1966; Ultmann, 1966), showing male predominance and increased incidence in the third and fourth decades, but indicating that the disease occurs at all ages. While presenting symptoms were similar to those reported by the above-mentioned investigators, 72% of cases in the present study exhibited generalised peripheral lymphadenopathy on admission. This finding was observed in 13·1–42·4% of cases by Meighan (1961), Westling (1965), Lukes *et al.* (1966), Peters *et al.* (1966), and Smithers (1967). Other investigators have not mentioned generalised peripheral lymphadenopathy as an important presenting sign (Jackson and Parker, 1947; Lumb, 1954; Craver and Miller, 1966; Ultmann *et al.*, 1966). The incidence of anaemia on admission was also much higher in the present study. Disease localised to one lymph node group was uncommon. These facts explain the shorter survival of patients in the present study, as patients with the disease localised to one lymph node group tend to have much better prognosis (Peters *et al.*,

1966). Cases of Hodgkin's disease have been classified into paragranuloma, granuloma and sarcoma, using the criteria of Jackson and Parker (1947). Only one case of paragranuloma was encountered, indicating marked paucity of this type. The majority of cases belonged to the granuloma group (85·6%). It was observed that nodular sclerosis (Lukes, 1963, 1966) was uncommon, and using the classification of Lukes (1966) the majority of cases would belong to the mixed cellularity group. The cases of Hodgkin's sarcoma, which correspond to the lymphocytic depletion group in the classification of Lukes *et al.* (1966), exhibited worse prognosis and on the whole more widely disseminated disease, thus showing good correlation between the more atypical histology and poor prognosis. The equal sex incidence in the sarcoma group was in agreement with the findings of Jackson and Parker (1947). There was no marked difference in age at the time of diagnosis between cases of sarcoma and granuloma in the present study. This is in contrast to the findings of Jackson and Parker (1947), who observed Hodgkin's sarcoma mainly in elderly patients. In the present study, children of 15 years or younger comprised 7·9% of cases. This is in agreement with reports from Europe and North America (Jackson and Parker, 1947; Lumb, 1954; Peters and Middlemiss, 1958; Westling, 1965). In Kenyan Africans Linsell (1967) found that 46% of cases of Hodgkin's disease occurred in children. A high proportion of children among cases of Hodgkin's disease has also been reported from Peru (Solidoro *et al.*, 1966), Ceylon (Cooray and Perera, 1966), and from Lebanon (Azzam, 1966). Solidoro *et al.* (1966) also stated that histologically a high proportion of their cases in children exhibited a picture of Hodgkin's sarcoma. All the cases in children in the present study showed a pattern of Hodgkin's granuloma, and the prognosis was relatively good. This is in accordance with other recent reports (Pitcock *et al.*, 1959; Aisenberg, 1964; Peters *et al.*, 1966).

The age and sex incidence of patients with lymphosarcoma were similar to other reported series (Jackson and Parker, 1947; Lumb, 1954; Rosenberg *et al.*, 1961) as was the clinical presentation, except for the fact that generalised peripheral lymphadenopathy was much more common in the present study. Anaemia was more frequent. Localised involvement of the stomach or small intestine was also relatively more common. There was good correlation between prognosis and histology when this group was subdivided into lymphocytic and lymphoblastic types which is in agreement with the large series reported by Rosenberg *et al.* (1961).

Reticulum cell sarcoma was not common in the present study. The age and sex incidence differed from those observed in other reported series (Jackson and Parker, 1947; Lumb, 1954; Meighan, 1961; Symmers, 1966) as a larger number of patients was under 40 years of age, and the sex incidence was equal. Generalised peripheral lymphadenopathy, anaemia, and hepatosplenomegaly on admission were more common in the present study. Survival in this group was shorter and prognosis poorer than in other types of malignant lymphoma. This is in agreement with other reported series (Jackson and Parker, 1947; Lumb, 1954; Hurst and Meyer, 1961; Rosenberg *et al.*, 1961; Symmers, 1966).

A paucity of patients with giant follicular lymphoma is evident from the present study. This is similar to reports from parts of Africa populated by Negroes (Camain and Lambert, 1964; Davies, 1964; Edington and Maclean, 1964; Oettlé, 1964; Wright and Roberts, 1966), as well as those from Asia (Chae Koo Lee *et al.*, 1965; Desai *et al.*, 1965). The features of cases in the present study were similar to those described in other series (Lumb, 1954; Hurst and Meyer, 1961; Rosenberg *et al.*, 1961; Dorfman, 1964; Symmers, 1966).

I would like to thank Professor G. Bras, Director of the Jamaica Cancer Registry, for encouragement and help.

This study formed part of a thesis submitted to the University of Sheffield for the degree of Doctor of Medicine.

### REFERENCES

AISENBERG, A. C.—(1964) *New Engl. J. Med.*, **270**, 508, 565, 617.
AZZAM, S. A.—(1966) *Cancer Res.*, **26**, 1202.
BRAS, G., WATLER, D. C. AND ASHMEADE-DYER, A.—(1965) *Br. J. Cancer*, **19**, 681.
BURKITT, D.—(1958) *Br. J. Surg.*, **46**, 218.
BURKITT, D. and O'CONOR, G. T.—(1961) *Cancer, N.Y.*, **14**, 258.
CAMAIN, R. AND LAMBERT, D.—(1964) Symposium on Lymphoreticular Tumours in Africa. Edited by F. C. Roulet. Basel/New York (S. Karger).
CHAE KOO LEE, SANG IN KIM AND JE G. CHI—(1965) *Seoul J. Med.*, **6**, 77.
COORAY, G. H. AND PERERA, R.—(1966) *Br. J. Cancer*, **20**, 1.
CRAVER, L. F. AND MILLER, D. G.—(1966) 'The Malignant Lymphomas'. American Cancer Society Publication.
DAVIES, J. N. P.—(1964) Symposium on Lymphoreticular Tumours in Africa. Edited by F. C. Roulet. Basel/New York (S. Karger).
DESAI, P. B., MEHER-HOMJI, D. R. AND PAYMASTER, J. C.—(1965) *Cancer, N.Y.*, **18**, 25.
DORFMAN, R. F.—(1964) Symposium on Lymphoreticular Tumours in Africa. Edited by F. C. Roulet. Basel/New York (S. Karger).
EDINGTON, G. M. AND MACLEAN, C. M. U.—(1964) Symposium on Lymphoreticular Tumours in Africa. Edited by F. C. Roulet. Basel/New York (S. Karger).—(1965) *Br. J. Cancer*, **19**, 471.
EL-GAZAYERLI, M., KHARADLY, M., KHALIL, H., GALAL, R., RIAD, W. AND EL-GAZAYERLI, M. M.—(1964) Symposium on Lymphoreticular Tumours in Africa. Edited by F. C. Roulet. Basel/New York (S. Karger).
GALL, E. A. AND MALLORY, T. B.—(1942) *Am. J. Path.*, **18**, 381.
GILLIAM, A. G.—(1953) *Blood*, **8**, 693.
GOLDMAN, L. B.—(1940) *Am. J. med. Ass.*, **114**, 1611.
HILTON, G. AND SUTTON, P. M.—(1932) *Lancet*, i, 283.
HURST, D. W. AND MEYER, O. O.—(1961) *Cancer, N.Y.*, **14**, 753.
JACKSON, H. JR. AND PARKER, F. JR.—(1947) 'Hodgkin's Disease and Allied Disorders'. New York (Oxford University Press).
LINSELL, C. A.—(1967) *Br. J. Cancer*, **21**, 465.
LUKES, R. J.—(1963) *Am. J. Roentg.*, **90**, 944.—(1966) *Cancer Res.*, **26**, 1311.
LUKES, R. J., BUTLER, J. J. AND HICKS, E. B.—(1966) *Cancer, N.Y.*, **19**, 317.
LUMB, G.—(1954) 'Tumours of Lymphoid Tissue'. Edinburgh (E. & S. Livingstone Ltd.).
MCMAHON, B.—(1966) *Cancer Res.*, **26**, 1189.
MEIGHAN, S. S.—(1961) *Can. med. Ass. J.*, **84**, 631.
O'CONOR, G. T.—(1961) *Cancer, N.Y.*, **14**, 270.
OETTLÉ, A. G.—(1964) Symposium on Lymphoreticular Tumours in Africa. Edited by F. C. Roulet. Basel/New York. (S. Karger).
PETERS, M. V., ALISON, R. E. AND BUSH, R. S.—(1966). *Cancer, N.Y.*, **19**, 308.
PETERS, M. V. AND MIDDLEMISS, K. C. H.—(1958) *Am. J. Roentg.*, **79**, 114.
PITCOCK, J. A., BAUER, W. C. AND MCGAVRAN, M.—(1959) *Cancer, N.Y.*, **12**, 1043.
RAPPAPORT, H., WINTER, W. J. AND HICKS, E. B.—(1956) *Cancer, N.Y.*, **9**, 792.

ROSENBERG, S. A., DIAMOND, H. D., JASLOWITZ, B. AND CRAVER, L. F.—(1961) *Medicine, Baltimore*, **40**, 31.
RUTTNER, J. R. AND WINTERHALTER, K. H.—(1966) Symposium on Lymphoreticular Tumours in Africa. Edited by F. C. Roulet. Basel/New York. (S. Karger).
SMITHERS, D. W.—(1967) *Br. med. J.*, ii, 263, 337.
SOLIDORO, A., GUZMAN, C. AND CHANG, A.—(1966). *Cancer Res.*, **26**, 1204.
STEINER, P. E.—(1954) 'Cancer: Race and Geography'. Baltimore (The Williams and Wilkins Co.)
SYMMERS, W. ST. C.—(1966) 'Systemic Pathology'. Edited by G. Payling Wright and W. St. C. Symmers. London (Longmans, Green & Co. Ltd.)
UDDSTROMER, M.—(1934) *Acta tuberc. scand.* Suppl. 1.
ULTMANN, J. E.—(1966) *Cancer, N.Y.*, **19**, 297.
ULTMANN, J. E., CUNNINGHAM, J. K. AND GELLHORN, A.—(1966) *Cancer Res.*, **26**, 1047.
WALLHAUSER, A.—(1933) *Archs Path.*, **16**, 522.
WESTLING, P.—(1965) *Acta Radiol.* Suppl. 245.
WRIGHT, D. H. AND ROBERTS, M.—(1966) *Br. J. Cancer*, **20**, 469.